# Antioxidant Effects of Myo-Inositol Improve the Function and Fertility of Cryopreserved Boar Semen

**DOI:** 10.3390/antiox12091673

**Published:** 2023-08-26

**Authors:** Rana Osman, Seongju Lee, Areeg Almubarak, Jae-Ik Han, Il-Jeoung Yu, Yubyeol Jeon

**Affiliations:** 1 Department of Theriogenology and Reproductive Biotechnology, College of Veterinary Medicine, Jeonbuk National University, Iksan 54596, Republic of Korea; rana09209@jbnu.ac.kr (R.O.); bart0522@jbnu.ac.kr (S.L.); areegalmubarak@jbnu.ac.kr (A.A.); iyu@jbnu.ac.kr (I.-J.Y.); 2 Department of Veterinary Medicine and Surgery, College of Veterinary Medicine, Sudan University of Science and Technology, P.O. Box 204, Hilat Kuku, Khartoum North 11111, Sudan; 3 Laboratory of Wildlife Medicine, College of Veterinary Medicine, Jeonbuk National University, Iksan 54596, Republic of Korea; jihan@jbnu.ac.kr

**Keywords:** cryopreservation, boar semen, myo-inositol, in vitro fertilization, apoptosis, ROS

## Abstract

During cryopreservation, sperm undergoes structural and molecular changes such as ice crystal formation, DNA fragmentation, and reactive oxygen species (ROS) production, leading to decreased sperm quality after thawing. Antioxidants play a crucial role in preventing these damages, both in vivo and in vitro. One potent antioxidant is myo-inositol, known for its protective effects on sperm against ROS. This study aimed to investigate the protective effect of myo-inositol on cryopreserved boar semen. The semen was diluted, cooled, and cryopreserved using a BF5 extender. It was then divided into five groups: control and different concentrations of myo-inositol (0.5, 1, 1.5, and 2 mg/mL). The post-thaw evaluation included assessments of motility, viability, acrosome integrity, mitochondrial membrane potential (MMP), caspase activity, gene expression, ROS levels, apoptosis, and IVF with treated semen. Results showed that myo-inositol at 0.5 mg/mL improved motility, acrosome integrity, and fertilization ability. It also reduced the expression of pro-apoptotic genes and increased *SMCP* expression. Lower concentrations also demonstrated improved viability and reduced apoptosis and ROS levels. In conclusion, myo-inositol treatment during cryopreservation improved sperm quality, reduced apoptosis and ROS levels, and enhanced fertility rates in boar semen.

## 1. Introduction

Cryopreservation is an essential procedure for the long-term preservation of semen. During this process, multiple changes occur, such as fluctuations in pH, osmotic changes, and energy depletion. These changes then lead to chemical, mechanical, and structural changes, as well as excessive reactive oxygen species (ROS) production, and consequently, cryoinjuries occur [1,2].

In a normal state, ROS are produced in small amounts to maintain normal sperm functions, which include facilitating vital intracellular signaling cascades for appropriate sperm functions such as maturation, hyperactivation, and capacitation, as well as the acrosome reaction (AR). This ROS ends up being transferred into harmless by-products by the natural antioxidant system found in semen or seminal plasma [3]. However, the massive increase in ROS production is due to an imbalance between oxidants and antioxidants, and thus oxidative stress occurs. Boar sperm have a substantial amount of intracellular superoxide dismutase (SOD) for scavenging O_2_^−^ and for rapid dismutation of O_2_^−^ to H_2_O_2_, but an extremely low level of catalase, which converts H_2_O_2_ to water and oxygen. This system is able to protect sperm during cooling but not cryopreservation due to the lack of catalase and exhaustion of other antioxidants. During freezing and thawing, there was an increase in the production of H_2_O_2_, and this is probably the primary source of oxidative damage during this process. An increase in ROS production causes extensive cell damage, including morphological defects, lipid peroxidation, DNA fragmentation, decreased fusion with the oocyte, and compromised pregnancy after in vitro fertilization [4]. Sperm tolerance to cryopreservation damage is related to membrane constituents; thus, high lipid content leads to massive damage by ROS produced during this process [5]. The membrane structure of boar sperm is rich in polyunsaturated fatty acids and poor in cholesterol, which renders it more susceptible to damage by reactive oxygen species due to excessive oxidative stress. As a result, a series of changes occur, starting with the destabilization of the plasma membrane, which loses its selective permeability, allowing the entry of Ca^2+^, bicarbonate, and media components into the gamete. This disrupts homeostasis, facilitates the degradation of proteins and mRNAs, and worsens fertilization rates [6,7]. Indeed, 40 to 50% of cryopreserved boar semen died after the freeze–thaw process due to several changes that occurred during this process. The conception rate of cryopreservation semen is lower than that of fresh semen, which delayed its use for AI in boars; only 1% of cryopreserved semen was used compared to 99% of fresh semen [8,9,10]. To improve the outcomes of cryopreserved semen, numerous studies have attempted to identify new semen additives that reduce the deleterious effects of cryopreservation and improve the quality of cryopreserved semen [11].

An antioxidant is one of the freezing–thawing semen additives that have ameliorative effects on semen quality by maintaining the balance between reductive and oxidative action, which improves sperm motility, membrane integrity, and fertilization ability [12,13]. Either found in plant extract or synthetic form, both forms have been supplemented on freezing–thawing media and reveal beneficial effects, such as extracts of the *Lamiaceae* that contain rosmarinic acid with known beneficial effects on frozen-thawed boar sperm motility, viability, acrosome integrity, and reduced lipid peroxidation and DNA oxidation. Extract of *Camellia sinensis* (green tea extract) contains polyphenols that improve frozen-thawed sperm viability, reduce lipid peroxidation, and increase monospermic and blastocyst formation. Grapes contain multiple antioxidants such as myo-inositol and resveratrol, which are known to enhance frozen-thawed sperm parameters including mitochondrial membrane potential, decreasing ROS, and apoptosis [9,14,15]. Synthetic antioxidants such as butylate hydroxytoluene, cysteine, and ascorbic acid reveal inhibitory effects on frozen-thawed boar semen [16].

Myo-inositol is a compound that is naturally extracted from various organisms. It is primarily found in plants and fungi; it is present in many foods such as fruits, seeds, and nuts; and it is also produced within the human body [17]. It plays an important role in cell growth and development, mediates transduction in response to neurotransmitters and hormones, and acts as a secondary mediator for regulating FSH, which is responsible for Sertoli cell numbers and function. Additionally, it reduces LH secretion and increases inhibin B secretion, which reduces FSH production. It also regulates and increases intracellular Ca^2+^ levels, osmoregulation, and ATP production, thus regulating sperm motility and capacitation, improving acrosome reactions, improving zona pellucida binding and fertilization ability, stimulating oxidative metabolism, activating the Akt protein that induces sperm maturation, and increasing phosphorylation of BCL-2 [14,18,19,20,21]. Naturally, it is found in the seminal plasma of domestic animals, including bulls, guinea pigs, monkeys, rabbits, rams, and boars. However, boar semen has the highest concentration of myo-inositol (2.4 g per 100 g of fresh weight) compared to the concentration found in animal and plant tissues [22,23]. Multiple studies have investigated the effects of different concentrations of myo-inositol on in vitro sperm parameters in humans and animals, and doses ranging from 1 mg/mL to 2 mg/mL reveal a significant increase in sperm parameters and reduced ROS and apoptosis levels after myo-inositol supplementation [15,18,24,25,26]. Hence, since no previous study investigated in vitro supplementation of myo-inositol on freezing–thawing media of boar, we investigated its effects on after-thawing boar sperm parameters and fertilization ability.

## 2. Materials and Methods

### 2.1. Chemicals

All reagents were purchased from Sigma-Aldrich (St. Louis, MO, USA), unless otherwise mentioned within the article. 

### 2.2. Semen Process, Extender Preparation, and Myo-Inositol Supplementation

Semen was collected from fertile Duroc boars from the KPG AI Center, Korea; 60 ejaculates from different boars were used in this study. Samples were collected using the gloved-hand method, and sperm-rich fractions of ejaculates with >75% motile and 80% morphologically normal spermatozoa were used in this study. Eligible samples were diluted (2.5 × 109 ± 0.5/90 mL) in BTS. The diluted semen was cooled and maintained at 17 °C and transported to the laboratory within 1 h of collection and dilution. Extended semen was received and distributed in a 15 mL falcon tube and kept at 17 °C for 2 h, then processed. A tris-egg yolk extender was prepared according to a previously described method [27] as follows: TES: 12 g/L; Trizma base: 2 g/L; D(+) glucose: 32 g/L; OEP (Minitube, Tiefenbach, Germany): 0.7% (vol:vol); Gentamycin sulfate: 0.02 g/L; egg yolk: 20% (vol:vol); with additional glycerol: 4% for extender 2; and all chemicals were dissolved in high-purity water from proGen, Genetrone Biotech, Jeonju, Republic of Korea. The extender was divided into five parts, and different concentrations of myo-inositol were added as follows: 0, 0.5, 1, 1.5, and 2 mg/mL.

### 2.3. Semen Cryopreservation and Thawing Procedure

Motility was evaluated after 2 h of incubation, and a motility of >80% was used. Semen was cryopreserved according to a modified two-step protocol that was previously described [28], as follows: semen was centrifuged at 17 °C for 7 min at 520× *g*, washed with Beltsville thawing solution (BTS), and centrifuged again. The supernatant was removed, and semen was added to extender 1 at a concentration of 2 × 10^8^ sperm/mL and incubated at 4 °C for 1 h. Extender 2 was then added to extender 1 at a ratio of 1:1, and semen was mixed with the extender. The mixture was placed in a 0.5 mL semen straw (Minitube, Tiefenbach, Germany) and equilibrated at 4 °C for 25 min. The semen straw was placed over a 4 cm liquid nitrogen surface for 20 min and then inserted into LN2 for storage. After at least 24 h, frozen semen was thawed at 37.5 °C for 25 s. Thawed semen was diluted 1:4 (vol:vol) with BTS, and the pertinent parameters were evaluated.

### 2.4. Assessment of Semen Motility and Kinematics after the Freezing–Thawing Process

Thawed semen motility was evaluated using computer-assisted sperm analysis (Sperm Class Analyzer, Microptic, Barcelona, Spain). Briefly, 2 µL of diluted thawed sperm were placed into a chamber (Leja, Nieuw-Vennep, The Netherlands) on a warm plate at 38 °C, and five fields for each group were evaluated. The adjustment of parameters is as follows: area: 10–80 µm^2^ (min–max); static: <10 µm/s; drafting: 0; slow-medium: 25 µm/s; rapid: >45 µm/s; progressive STR: >45; connectivity: 11 pixels; VAP points: 5 pixels; head point: 10–100; and frame rate: 25 frames per second. Consecutive parameters were evaluated, including total sperm motility (TM%), progressive motility (PM%), rapid progressive motility (RPM), and medium progressive motility (MPM). Additionally, sperm kinematics were assessed, including curvilinear velocity (VCL, µm/s), average path velocity (VAP, µm/s), straight linear velocity (VSL, µm/s), straightness (STR%, VSL/VAP), and linearity (LIN%, VSL/VCL).

### 2.5. Assessment of Semen Viability after the Freezing–Thawing Process

Viability was evaluated using the LIVE/DEAD™ Sperm Viability Kit (ThermoFisher, Waltham, MA, USA) according to a previously described method [29,30]. In brief, 50 µL of diluted thawed semen was placed in an Eppendorf tube, and then 5 µL of 1 mM SYBR-14 stain was added to the thawed semen and incubated for 5 min in the dark. After incubation, 5 µL of 2.4 mM propidium iodide (PI) was added to the semen and incubated for an additional 5 min. Two clean glass slides were prepared, and 10 µL of stained semen was smeared and examined under a fluorescence microscope (Axio, Carl Zeiss, Goettingen, Germany). A total of 200 sperm cells were counted from the two smears using two filters: 488/516 nm for SYBR-14, where green fluorescence indicates live sperm, and 535/617 nm for PI red head, which indicates dead sperm. 

### 2.6. Assessment of Acrosome Integrity after the Freezing–Thawing Process

According to Yu’s method, a combination of two strains of *Pisum sativum* agglutinin (PSA) were combined with fluorescein isothiocyanate (FITC) [31]. A clean glass slide was prepared with ten microliters of post-thaw diluted semen smeared onto it and dried. The smear was fixed with absolute alcohol and dried. Approximately 20 to 30 µL of the stained mixture (100 mg/mL) was added to each slide, covered with parafilm (Bemis, Chicago, IL, USA), and incubated in the dark for 20 min. After incubation, the slides were immersed in distilled water to remove the parafilm, dried, and examined using fluorescence microscopy (Axio, Carl Zeiss Microscopy, White Plains, NY, USA). At least 200 sperm cells per slide were counted. Strong green fluorescence in the sperm acrosome was considered intact, whereas others were considered nonintact acrosomes.

### 2.7. Assessment of Caspase after the Freezing–Thawing Process

Caspase activity was detected using a CaspGLOW™ Red Active Caspase Staining Kit and a Fluorescein Active Caspase Staining Kit (Bio Vision, Milpitas, CA, USA) in accordance with the manufacturer’s instructions. The detailed methodology is as follows: 0.5 mL of thawed semen was kept in a 15 mL falcon tube, diluted with 3.2 mL of DBPS, and 300 µL of the mixture was transferred to an Eppendorf tube. Thereafter, 1 µL of sulforhodamine (Red-VAD-FMK^®^, Sigma-Aldrich, St. Louis, MO, USA) was mixed in, and it was incubated at 37 °C for 20 min in the dark. The billet was centrifuged for 5 min at 860 g, the supernatant was discarded, and then the billet was resuspended in 500 mL of Dulbecco’s phosphate-buffered saline (DPBS). Following this, 10 µL was placed on the slide, cover slipped, and examined under a fluorescence microscope (Axio, Carl Zeiss, Goettingen, Germany) using a rhodamine filter. A total of 200 sperm cells were counted. The positive cells showed strong red fluorescence in the middle piece and tail, and the negative cells showed red without strong fluorescence [32].

### 2.8. Assessment of Mitochondrial Membrane Integrity after the Freezing–Thawing Process

The mitochondrial membrane integrity was assessed using a combination of rhodamine 123 (Molecular Probes, Eugene, OR, USA) and PI [33]. An amount of 250 µL diluted semen was mixed with 5 µL (0.01 mg/mL) rhodamine solution and 5 µL PI (2.4 mM) and incubated in the dark for 15 min. Smears were prepared and examined under a fluorescence microscope (Axio, Carl Zeiss, Goettingen, Germany), with a total of 200 sperm merged between FITC and rhodamine filters. Strong green fluorescence in the middle sections was considered positive, whereas sperm without strong green fluorescence in the middle were considered negative.

### 2.9. Assessment of Gene Expression after the Freezing–Thawing Process by One-Step Relative Quantitative PCR

To assess gene expression, the total RNA for the control and myo-inositol-treated groups was prepared using the TRI method [34] and TRIZOL reagent^®^ (Molecular Research Center, Inc., Cincinnati, OH, USA) in accordance with the manufacturer’s instructions. Briefly, after thawing at 37 °C for 25 s, spermatozoa were washed twice with DPBS (centrifugation at 160× *g* for 2 min), and the supernatant was removed. The TRIZOL reagent was added, mixed well with the sample, and incubated for 5 min at room temperature. Then chloroform was added at a rate of 1:5 TRIZOL, mixed well, and incubated for another 5 min. Then it was centrifuged at 1600× *g* at 4 °C for 10 min. Then take 30 µL of supernatant and mix with 30 µL of isopropanol and 2 µL of glycogen (BIOSOLUITION Suwon-si, Republic of Korea). The concentration and purity of RNA were measured (Epoch™ Microplate spectrophotometer, Agilent, Santa Clara, CA, USA). Total RNA from all samples was equalized to the same concentration, and quantitative real-time PCR (qPCR) was conducted to assess transcription abundance (primers are listed in Table 1). qPCR was used to analyze the mRNA expression of the following genes: Bcl-2-associated X protein (BAX), BCL2 Apoptosis Regulator (BCL2), Reactive Oxygen Species Modulator (ROMO1), Spermine Oxidase (SMOX), NADPH Oxidase 5 (NOX5), and Sperm Mitochondria Associated Cysteine Rich Protein (SMCP). These were assisted by using the One-Step TB Green^®^ Prime Script™ RT-PCR Kit II (Takara, Bio USA, Inc., Mountain View, CA, USA) and an ABI 7500 real-time PCR system (Applied Biosystems, Beverly, MA, USA). The total volume of the reaction mixture was 20 µL, consisting of the following: 2 µL RNA, 10 µL TB buffer, 0.8 µL enzyme mix, 0.8 µL forward primer, 0.8 µL reverse primer, 0.4 µL ROX dye II, and 5.2 µL nuclear-free water. The PCR conditions were as follows: stage 1, reverse transcription: 5 min at 42 °C and then 10 s at 95 °C; stage 2, PCR reaction: 40 cycles of 5 s at 95 °C and 34 s at 56 °C; and stage 3, dissociation. GAPDH (glyceraldehyde 3-phosphate dehydrogenase) was used as an indigenous control, while the value of gene expression for the control group was set at 1. The gene quantities were determined by using the equation n R = 2 − (∆Ct sample − ∆Ct control). Table 1 illustrates the primers used for qPCR.

### 2.10. Assessing In Vitro Fertilization Ability

#### 2.10.1. Oocyte Maturation

After the ovaries arrived at the lab in physiological saline, the initial temperature was evaluated at 35–37 °C. The medium-sized follicle content was aspirated using 18-gauge hypodermic needles attached to a 10 mL syringe. The collected oocytes were washed twice with Hep-buffered Tyrode’s medium (TLH) and transferred to a petri dish containing TLH, where at least two layers of cumulus and granulated cytoplasmic oocytes were collected. Every 60 oocytes were cultured in IVM medium TCM-199 (Invitrogen, Waltham, MA, USA) supplemented with cysteine, porcine follicular fluid, sodium pyruvate, epidermal growth factor (EGF), equine chronic gonadotropin, hCG, kanamycin, and insulin. Oocytes were incubated at 39 °C and 5% CO_2_ for 22 h with hormones and 20 h without hormones [35].

#### 2.10.2. In Vitro Fertilization

For the evaluation of IVF, two groups of cryopreserved semen were assessed: the control and myo-inositol-treated semen groups. In brief, mature oocytes were selected, washed twice with IVF medium (MTBM), and moved into fertilization drops containing 40 µL of MTBM covered with mineral oil (Thermofishers Scientific Solutions Co., Ltd., Waltham, MA, USA), each drop containing 15 oocytes, and each group containing at least 30 oocytes, which were then placed in the incubator. After thawing, the semen was washed twice with centrifugation at 520× *g* for 2 min, first with BTS and then with DPBS. The supernatant was discharged, and 200 µL of MTBM was added and mixed. Of this, 10 µL was taken for motility testing, and 10 µL was used for concentration determination. After complete concentration determination, the sperm concentration was adjusted to 5 × 10^6^ sperm/mL for fertilization. Each drop received 5 µL of the prepared sperm. The oocytes and sperm were co-incubated in a 5% CO_2_ incubator at 39 °C for at least 6 h, then the oocyte was denuded by pipetting to remove extra sperm around the oocytes [36].

#### 2.10.3. Embryo Culture

Following IVF, presumed zygotes were rinsed twice with Porcine Zygote Medium 3 (PZM-3) culture medium, and every 10 oocytes were placed in a PZM drop covered with mineral oil and incubated in two gas incubators (5% CO_2_ and 5% O_2_ at 39 °C). Cleavage was checked on Day 2, and blastocysts were checked on Day 7 [37]. At least three replicates were used to evaluate the cleavage rate and blastocyst percentage for the control and 0.5 mg/mL groups.

### 2.11. Assessment of Lipid Perodixation

Lipid peroxidation was assessed via a lipid peroxidation kit (MDA) (BioVision, Mountain View, CA, USA). Two experiment groups, the control and myo-inositol-treated groups, were tested in brie, and thawed semen was washed two times with PBS, then the pellet was mixed with 300 µL of MDA lysis buffer and 3 µL of BHT, mixed and homogenized in ice for 10 min, then centrifuged at 4 °C and 13,000× *g* for 10 min. A total of 200 µL of the supernatant was added to 600 µL of TBA in an Eppendorf tube. Six standers were prepared: 0, 1, 2, 4, 6, 8, and 10. The samples and standers were incubated for 1 h at 95 °C, then cooled on ice for 10 min. A modified centrifugation step was added for 1 min at 720× *g* for precipitate deprivation. In a non-treated cell culture plate containing 96 wells (SPL Life Science Co., Ltd., Pocheon, Republic of Korea), 200 µL of stander and sample were added to each well in duplicate and read in a spectrophotometer (Agilent, Santa Clara, CA, USA) at 532 nm. The result of the mean reading is an MDA level of nmol/2 × 10^7^.

### 2.12. Assessment of Sperm ROS after the Freezing–Thawing Process

To determine ROS levels, 2′,7′-dichlorodihydrofluorescein diacetate (H2DCFDA; Molecular Probes Inc.) and the following protocol were used [38]. In brief, a 0.5 mL straw from the control and myo-inositol-treated groups was thawed as previously described. Thereafter, thawed semen was centrifuged at 370× *g* for 3 min, after which the supernatant was discarded, and the belts were suspended in 500 µL of DPBS. This was repeated, and the belt was resuspended into 500 µL of DPBS and mixed well by pipetting. An amount of 50 µL of suspension was added to 950 µL of DBPS in a 5 mL FACS tube (FALCON). Then, 1 µL of 20 mM 2′,7′-dichlorodihydrofluoresceindiacetate (H2DCFDA DCF, Invitrogen, Carlsbad, CA, USA) and 1 µL of PI stock (2.4 mM) were added and incubated in the dark for 1 h. Samples were analyzed using a FACSCalibur flow cytometer (Becton Dickinson, San Jose, CA, USA). All non-sperm events were excluded from further analysis. The mean fluorescence intensity (MFI) and FL1 (DCF) signals were detected using a 530/30 nm bandpass filter. FL2 (PI) signals were detected using a 585/42 nm bandpass filter, and the MFI of DCF was measured to evaluate the intracellular mean H_2_O_2_ in the sperm population. Three results were obtained: PI +ve, which indicates dead sperm; PI +ve/H2CFDA +ve, which indicates viable sperm with high H_2_O_2_; and PI −ve/H2CFDA −ve, which indicates live sperm with low H_2_O_2_. The mean ratio between viable sperm with high H_2_O_2_ and total viable sperm evaluated as ROS levels was calculated.

### 2.13. Assessment of Sperm Apoptosis after the Freezing–Thawing Process

Apoptosis was assessed using a combination of FITC annexin and PI stain [39] in accordance with the manufacturer’s instructions for the FITC Annexin V apoptosis detection kit I (BD Biosciences, San Diego, CA, USA). After thawing, sperm from the control and myo-inositol-treated groups were centrifuged in 300× *g* for 3 min, and the supernatant was discarded. Then the sperm pellet was resuspended in 1×Annexin V binding buffer (0.1 M Hepes/NaOH (pH 7.4), 1.4 M NaCl, and 25 mM CaCl_2_). For a 1X working solution, dilute 1 part of the 10X Annexin V Binding Buffer with 9 parts of distilled water. A total of 20 μL of suspension was added to 980 μL of 1×Annexin V binding buffer in an Eppendorf tube. Next, in a 5 mL FACS tube (FALCON), 5 μL of Annexin V-FITC stock and 5 μL of PI (stock of 1 mg/mL) were added to a 100 μL suspension containing sperm under dark conditions. After incubation for 15 min, 400 μL of 1×Annexin V binding buffer was added, and the samples were assessed using a FACSCalibur^®^ flow cytometer (Becton Dickinson, Franklin Lakes, NJ, USA). For this assessment, the labeling patterns in the annexin (AN)/PI analysis were classified as follows: viable (AN−/PI−), viable but phosphatidylserine (PS) translocated (AN+/PI−), dead and PS translocated (AN+/PI+), and dead and late necrotic sperm (AN−/PI+). The apoptosis index was calculated as the ratio of AN+/PI− spermatozoa to total viable (PI−) spermatozoa.

### 2.14. Experimental Design

This experiment was designed to study the effects of myo-inositol supplementation prior to freezing on the post-thaw sperm parameters of boars. The experiment was conducted in two trays: first, to evaluate the effect of myo-inositol supplementation on post-thawing sperm parameters, including motility parameters (CASA), viability (SYBR14/PI), acrosome integrity (PSA/FITC), and MMP (Rh123/PI). All parameters were measured for the five experimental groups.

In the second part of the experiment, the effects of myo-inositol on sperm parameters including oxidative status, apoptosis, gene expression, and fertilization parameters in cryopreserved boar sperm were evaluated. Six tests were carried out. To evaluate the IVF outcome by cleavage on Day 2 and blastocysts on Day 7, the control and 0.5 mg/mL myo-inositol-treated groups were selected to evaluate fertilization parameters and gene expression levels. qPCR was used to analyze the transcription levels of selected genes.

Oxidative status was assessed by measuring ROS levels and lipid peroxidation in two experimental groups: control and 0.5 mg/mL myo-inositol. We used H2DCFDA and PI flow cytometer probes to measure ROS and a spectrophotometer kit to measure lipid peroxidation.

Thirdly, we assessed apoptosis in two ways: caspase activity using a spectrophotometric kit (CaspGLOW™, ThermoFisher, Waltham, MA, USA) for all treated groups and apoptosis index (FITC-Anexin V and PI staining) by flow cytometry for the control and myo-inositol-treated groups.

### 2.15. Statistical Analysis 

Each experiment was repeated at least three times. All experimental data were evaluated using SAS software (version 9.4; SAS Institute Inc., Cary, NC, USA). Sperm-related data (e.g., motility, kinematics, viability, acrosome integrity, caspase activity, and mitochondrial membrane integrity) were compared using one-way analysis of variance, followed by Duncan’s multiple range test. An unpaired two-tailed Student’s *t*-test was conducted to evaluate the data of the remaining experiments (gene expression, embryo development, lipid peroxidation, ROS, and apoptosis). The results are expressed as mean ± SEM, and a probability value of *p* < 0.05 was statistically significant.

## 3. Results

### 3.1. Myo-Inositol Supplementation Effects on Frozen-Thawed Sperm Motility and Kinematics

**Figure 1:**
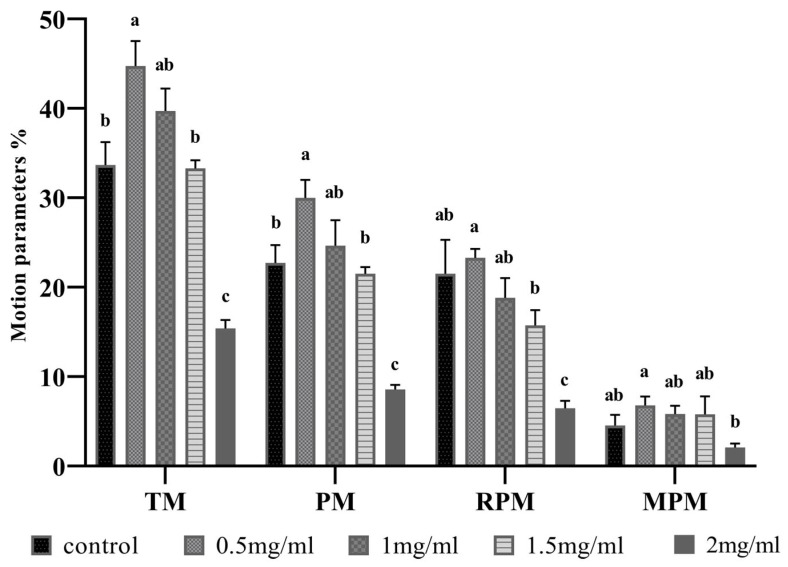
Effects of different concentrations of myo-inositol on sperm motility parameters after the freezing–thawing process. Control: 0 mg/mL, 0.5 mg/mL, 1 mg/mL, 1.5 mg/mL, and 2 mg/mL myo-inositol-treated groups; TM: total motility; PM: progressive motility; RPM: rapid progressive motility; MPM: medium progressive motility. Letters a, b, and c represent significant differences between groups (*p* < 0.05). Error bars show the SEM.

The effects of myo-inositol supplementation in semen extenders on boar sperm motility are shown in Figure 1. Compared to the control group, the 0.5 mg/mL myo-inositol-treated group showed a significant increase in total and progressive motility (*p* < 0.05). The rapid and medium-progressive motility of the myo-inositol-treated groups were not significantly different from that of the control group (*p* > 0.05). Between different myo-inositol concentrations, 0.5 mg/mL reveals a significant increase in TM, PM, and RPM, and 2 mg/mL reveals a significant decrease in all motion parameters compared to other myo-inositol groups.

Table 2 shows the effects of myo-inositol on sperm kinematics after freezing–thawing. The results illustrate five parameters, as follows: VLC, VAP, VSL, STR, and LIN. Compared to other myo-inositol concentrations, 2 mg/mL reveals a significant decrease in VLC and VAP, while there is no significant difference in parameters between other concentrations. For both straightness and linearity, there was no significant difference between all the experiment’s groups.

### 3.2. Effects of Myo-Inositol Supplementation on Sperm Viability

**Figure 2:**
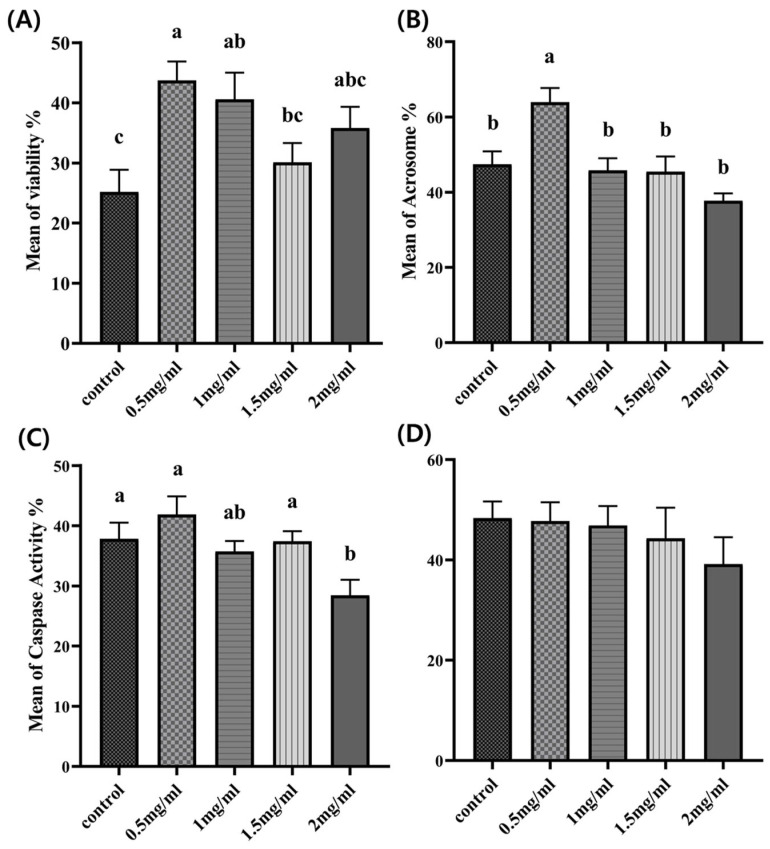
Effect of myo-inositol on sperm survival after the freezing–thawing process. (**A**) Semen viability; (**B**) acrosome integrity; (**C**) sperm caspase activity; and (**D**) sperm mitochondrial membrane potential. Control and 0.5 mg/mL, 1 mg/mL, 1.5 mg/mL, and 2 mg/mL myo-inositol-treated groups. Letters a, b, and c represent significant differences between the groups (*p* < 0.05). Error bars show the SEM.

Figure 2A depicts the effect of myo-inositol on sperm viability, showing that the number of viable sperm was significantly higher in both the 0.5 mg/mL and 1 mg/mL myo-inositol-treated groups (*p* < 0.05). The other treated groups had higher viability than the control group, but there was no statistical difference between them and the control group (*p* ≥ 0.05).

### 3.3. Effects of Myo-Inositol Supplementation on the Integrity of Frozen-Thawed Sperm Acrosomes

Figure 2B shows the effects of different myo-inositol concentrations on the acrosome integrity of cryopreserved boar semen. The acrosome integrity of sperm was evaluated after thawing, and the results revealed a significant increase in the number of sperm with intact acrosome membranes in the 0.5 mg/mL group compared to the control group. Among the myo-inositol-treated groups, the 0.5 mg/mL group also had significantly higher acrosome integrity than other concentrations.

### 3.4. Effects of Myo-Inositol Supplementation on Sperm Caspase

Figure 2C illustrates the effect of the myo-inositol-treated freezing medium on caspase activity. More than three independent replicates were used to evaluate caspase activity. The results revealed that the three highest concentrations of myo-inositol resulted in lower caspase activity than the control group, with significance in the 2 mg/mL group.

### 3.5. Effects of Myo-Inositol Supplementation on Sperm Mitochondrial Membrane Potential

Figure 2D shows the effects of myo-inositol supplementation in the freezing medium on the sperm mitochondrial membrane potential. A combination of PI and Rhodamine 123 staining was conducted to evaluate MMP, and the results showed no significant difference in mitochondrial membrane integrity among all experimental groups (*p* ≥ 0.05). 

### 3.6. Effects of Myo-Inositol Supplementation on Sperm Gene Expression

**Figure 3:**
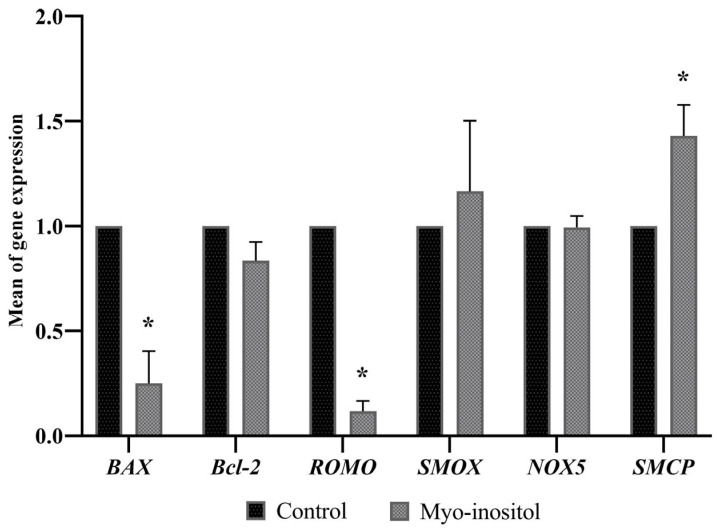
Effect of myo-inositol on gene expression after the freezing–thawing process. Control: 0 mg/mL myo-inositol; myo-inositol: 0.5 mg/mL myo-inositol. (*) Indicates a significant difference between the groups (*p* < 0.05). Error bars show the SEM. *BAX*: Bcl-2-associated X protein; *Bcl-2*: Bcl-2 apoptosis regulator; *ROMO1*: reactive oxygen species modulator 1; *SMOX*: spermine oxidase; *NOX5*: NADPH oxidase 5; *SMCP*: sperm mitochondria associated cysteine rich protein.

Based on the results of the prior experiments, the optimal treatment concentration was set at 0.5 mg/mL, and subsequent experiments were conducted with only the control and 0.5 mg/mL treatment groups. Real-time PCR was conducted for the control and 0.5 mg/mL myo-inositol-treated groups. Figure 3 shows the results of gene expression for the myo-inositol-treated group and the control group. *BAX*, a pro-apoptotic gene, showed a significant decrease in expression in the myo-inositol-treated group compared to the control group. The expression of the *Bcl-2* apoptosis regulator gene (*Bcl-2*), an anti-apoptotic gene, did not differ significantly from that in the control group. *ROMO1* gene expression levels were significantly lower in the myo-inositol group than in the control group; conversely, the *SMCP* gene, which is related to sperm motility, showed higher expression in the myo-inositol-treated group than in the control group. The *NOX5* and *SMOX* genes were not significantly different between the two groups.

### 3.7. Effect of Myo-Inositol-Treated Cryopreserved Semen on Fertilization Ability and Blastocyst Formation

**Figure 4:**
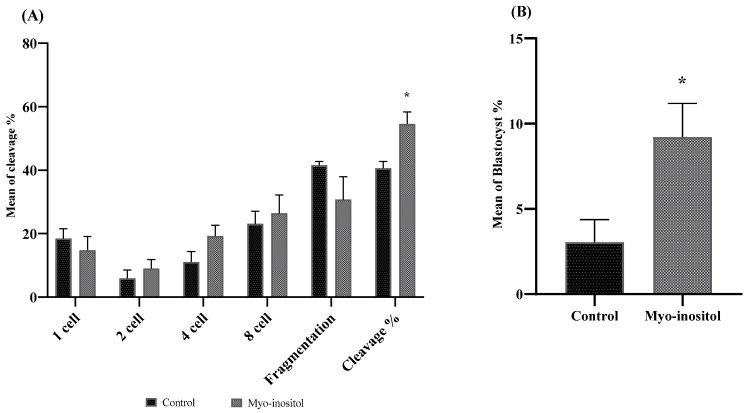
(**A**) Effect of myo-inositol-treated cryopreserved semen on the cleavage rate. (**B**) Effect of myo-inositol-treated cryopreserved semen on the blastocyst rate. Control: 0 mg/mL myo-inositol; myo-inositol: 0.5 mg/mL myo-inositol. (*) Indicates a significant difference between the groups (*p* < 0.05). Error bars show the SEM.

The effects of myo-inositol-treated semen were evaluated as previously described. For this test, the control and the myo-inositol-treated (0.5 mg/mL) groups were compared. The cleavage percentage was evaluated on Day 2, and blastocyst percentages were calculated on Day 7. Although there was no significant difference in cell number between the two groups, the myo-inositol-treated group demonstrated a significant increase in cleavage and blastocyst rates compared to the control group (*p* < 0.05), as shown in Figure 4.

### 3.8. Effects of Myo-Inositol Supplementation on Sperm Lipid Peroxidation

**Figure 5:**
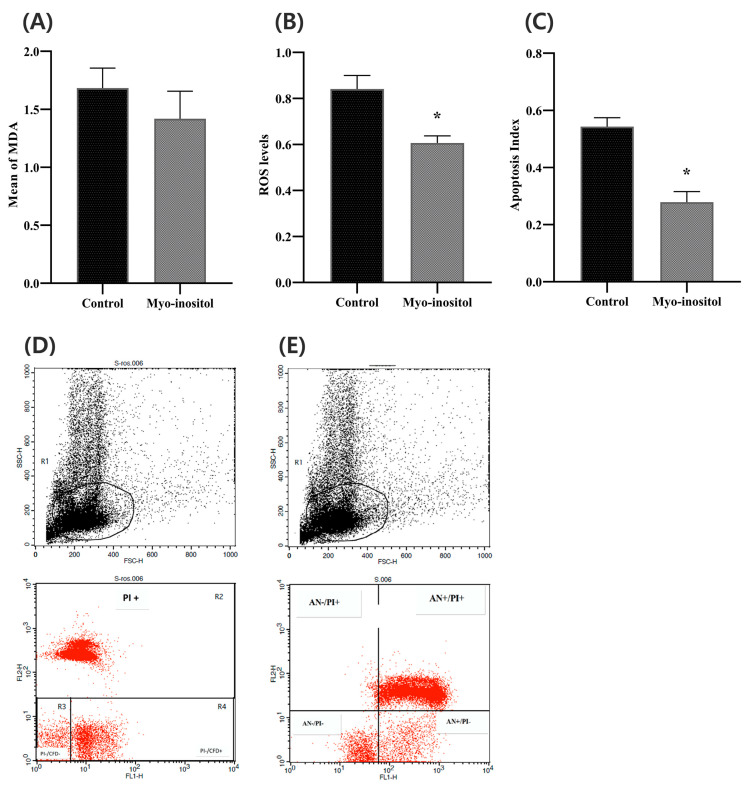
Effect of myo-inositol on sperm damage after the freezing–thawing process. (**A**) Sperm MDA levels; (**B**) sperm ROS; (**C**) sperm apoptosis; (**D**) control ROS; and (**E**) control apoptosis. Control: 0 mg/mL myo-inositol; myo-inositol: 0.5 mg/mL myo-inositol. (*) Indicates a significant difference between the groups (*p* < 0.05). Error bars show the SEM.

Figure 5A shows the effects of myo-inositol supplementation in the freezing medium on sperm lipid peroxidation. The mean of the spectrophotometer MDA levels was evaluated, and the results showed no significant difference in lipid peroxidation levels among the myo-inositol-treated group and the control group (*p* ≥ 0.05).

### 3.9. Effect of Myo-Inositol on ROS Levels in Cryopreserved Semen

Oxidative stress was evaluated using the reactive oxygen levels shown in Figure 5B,D. The results showed that the myo-inositol-treated group had significantly lower ROS levels than the control group. 

### 3.10. Effect of Myo-Inositol on Sperm Apoptosis

Flow cytometry revealed that myo-inositol positively affected the decrease in apoptosis levels after the cryopreservation. Figure 5C, E illustrates that the apoptotic sperm percentage in the myo-inositol-treated group was significantly lower than that in the control group.

## 4. Discussion

Boar sperm have weak defenses against ROS because of the low antioxidant concentration in seminal plasma. In addition to this, it contains a high concentration of polyunsaturated fatty acids (PUFA), which inhibit oxidative damage. During cryopreservation, an increase in free radicals leads to oxidative stress, which leads to structural and functional changes and finally apoptosis. Myo-inositol improves sperm quality, reduces ROS production, protects acrosome integrity and mitochondrial function, protects against cryodamage, and reduces apoptosis [40,41,42,43]. 

Sperm motility and viability are greatly reduced after thawing; this reduction is related to the factors mentioned previously [44,45]. This study reveals that supplementation of semen extenders with myo-inositol has a positive effect on sperm motility and viability. As shown in the results, a significant increase in TM, PM, and the viability of the 0.5 mg/mL myo-inositol group occurred. Additionally, 1 mg/mL significantly improves viability after the thawing process. Our results are consistent with previous studies that revealed that myo-inositol supplementation in thawed media improves the motility and viability of human and bovine sperm [46,47], and supplementation with myo-inositol in cryopreservation media improves motility and viability after thawing in human and dog sperm [15,25,26]. Myo-inositol has protective effects on sperm cells, and it improves ATP generation and increases cytosolic Ca++ concentration in the flagellum, which consequently improves sperm motility and viability [24,47,48,49,50]. On the other hand, in our study, the absolute values of several sperm kinematics were not improved, which disagreed with previous results in canine semen [26] that revealed a significant increase in kinematics in myo-inositol-supplemented groups. Our kinematic results may be due to two factors: first, variations in antioxidant behavior according to the species, as mentioned by [51], and second, the CASA system and boar semen, as mentioned in a previous study [52], increase FRs, which leads to increased kinematics but not increased TM% and PM%. In this study, we used 25 FRs.

Myo-inositol plays a role in protecting sperm acrosome integrity through osmoregulation and reducing ROS production [53]. In the present study, we found that myo-inositol improved sperm acrosomal integrity, as shown in Figure 3. This finding agrees with those of previous studies in dogs and goats, which revealed that supplementation of pre-frozen media with myo-inositol enhanced sperm acrosome integrity [26,54]. 

Our observation reveals negative effects of the highest myo-inositol concentration (2 mg/mL) on some boar sperm parameters. These results were not surprising. There was a previous study on dogs that revealed a significant decrease in motility, viability, and some kinematics in dogs, and the author refers to negative effects due to either disturbance in osmolarity or toxicity [26]. On the other hand, a recent study of boar sperm supplemented by 2–8 mg of myo-inositol in liquid preservation revealed 2 mg/mL as the optimal concentration [55]. Changes in membrane structure during freezing and thawing increase the sensitivity of sperm to high concentrations and may interfere with sperm metabolism, exerting these negative effects. 

Fertilization success is highly related to semen quality, which translates to the oxidative status of sperm and directly affects fertilization and blastocyst percentage [56,57]. Hence, cryopreservation of boar semen has a negative impact on fertility rate and embryo number [58]. The present study investigated the effect of myo-inositol on the fertilization ability of cryopreserved semen in the control and 0.5 mg/mL myo-inositol groups. The results indicated that supplementation of the semen extender with myo-inositol significantly improved cleavage and blastocyst formation compared to the control group (Figure 5). This result is consistent with a previous study in goats, which found that supplementation of the extender with myo-inositol improved cleavage and blastocyst rates [59]. Indeed, reducing ROS in sperm improves motility and morphology, which leads to improved fertility. This agrees with previous reports that recognized that myo-inositol reduced ROS production and improved acrosome integrity. Also, myo-inositol is one pre-source of phosphatidylinositol (PI), which improves sperm motility and sperm-zona pellucida binding [48,60,61,62,63]. An increase in H_2_O_2_ in sperm leads to a reduction in embryonic production and blastocyst number [64]. In our study, the control group revealed a higher concentration of H_2_O_2_, which may explain the low blastocyst numbers in the control group.

The freeze–thaw process is stressful to sperm, and during this process, sperm confront prolonged stress and increase ROS production, which leads to DNA fragmentation, changes in membranes, and cell apoptosis. Multiple studies report strong beneficial effects of myo-inositol on protecting sperm DNA and reducing ROS and apoptosis during cryopreservation [25,65,66]. Our study investigated the effect of different myo-inositol concentrations on boar sperm and evaluated oxidative stress and apoptosis parameters, including caspase activity, apoptosis index, ROS levels, and related genes, as shown in Figure 2C, Figure 3, and Figure 5B. The results revealed that treatment of boar semen with myo-inositol significantly decreased both the oxidative and apoptosis parameters as well as the pro-apoptotic gene expression levels of BAX and ROMO1, which are responsible for programmed cell death. In contrast, SMCP was significantly increased in the myo-inositol group compared to the control group. This result is consistent with previous studies in human and animal sperm, showing that myo-inositol treatment in cryopreserved semen decreased oxidative stress and apoptosis, increased the cryosurvival rate, and improved gene expression [24,26,54,65,67,68]. 

Reactive oxygen species (ROS) damage is a well-known occurrence in boar sperm cells since the presence of polyunsaturated fatty acids (PUFA) renders the sperm membranes more susceptible to oxidative damage. Lipid peroxidation caused by ROS leads to increased apoptosis [18,69]. Our study investigated the effects of myo-inositol on malondialdehyde (MDA) levels in both a control group and a group treated with 1 mg/mL of myo-inositol and found no significant differences between the two groups. There was, however, a variation in myo-inositol effects on MDA in different species, such as a significant decrease in bulls (3 mg/mL), goats (5 µM), and humans (2 mg/mL). A non-significant effect was found in rams (10 mM) with an extender containing skimmed milk and an extender containing sodium citrate, though the same concentration of myo-inositol was significant for a tris extender [25,54,70,71,72]. The behavior of antioxidants depends on several factors, such as species, concentration, and extender type [51], which may explain the variation in our results. 

## 5. Conclusions

In conclusion, supplementation of the semen extender with myo-inositol improved boar sperm parameters after the freeze–thaw process, including motility, viability, acrosome integrity, decreased apoptosis, oxidative state-related genes, reduced ROS levels, and improved fertilization ability and blastocyst rate. Our study recommendation is to use 0.5 mg/mL of myo-inositol as a supplement to the boar semen extender used for AI. To fully understand the benefits of myo-inositol on boar semen, further studies are needed to investigate its impact on embryo quality (both in vivo and in vitro) and lipid peroxidation.

## Figures and Tables

**Table 1:** Primer sequence used for the gene expression analysis.

Gene	Primer Sequence	Product Size (bp)	Accession Number
*Bcl-2*	F:TGGTGGTTGACTTTCTCTCC R: ATTGATGGCACTAGGGGTTT	752	NM_214285.1
*BAX*	F:CAGCTCTGAGCAGATCATGA R: TTGAGACACTCGCTCAACTT	833	XM_003127290.5
*GAPDH*	F:CTCTGGCAAAGTGGACATTG R: CATTGATGACAAGCTTCCCG	1341	NM_001206359.1
*ROMO1*	F: GAAGATGGGCTTTGTGATGG R: ATAGTACATGGGCTGGGACT	401	NM_001097462.2
*SMOX*	F: TGGAAGAGACAACTGATGGG R: CATGGTTATGGTCACCCTCA	2034	NM_001185170.1
*SMCP*	F: AGTGCACCTGCCTGAATAAG R: CCTACTTGTTTGGCTGCTTC	704	NM_001008685.1
*NOX5*	F: TTCTTCGCCCTCTTTGACTT R: CAGTCAAAGTTGAGGCACTG	8874	XM_021100544.1

F: forward; R: reverse; *BAX*: Bcl-2-associated X protein; *Bcl-2*: Bcl-2 apoptosis regulator; *ROMO1*: reactive oxygen species modulator 1; *SMOX*: spermine oxidase; *NOX5*: NADPH oxidase 5; *SMCP*: sperm mitochondria-associated cysteine-rich protein.

**Table 2:** Effects of different concentrations of myo-inositol on sperm kinematics.

Kinematic	Control	0.5 mg/mL	1 mg/mL	1.5 mg/mL	2 mg/mL
VLC	59.2 ± 5.98 ^a^	54.53 ± 1.8 ^ab^	50.5 ± 1.4 a^ab^	48.4 ± 4.7 ^ab^	43.35 ± 1.9 ^b^
VAP	48.2 ± 3.11 ^a^	43.56 ± 1.18 ^ab^	39.81 ± 1.88 ^bc^	39.4 ± 3.47 ^bc^	35.3 ± 1.52 ^c^
VSL	40.5 ± 2.27 ^a^	35.26 ± 0.96 ^ab^	31.85 ± 1.45 ^b^	33.18 ± 3.62 ^ab^	29.91 ± 2.21 ^b^
STR	72.4 ± 4.74 ^a^	70.83 ± 0.70 ^a^	70.85 ± 2.80 ^a^	74.57 ± 1.35 ^a^	72.25 ± 3.51 ^a^
LIN	59.0 ± 5.45 ^a^	56.28 ± 0.72 ^a^	56.63 ± 2.47 ^a^	60.95 ± 1.53 ^a^	58.38 ± 2.63 ^a^

Mean ± SEM. VCL: curvilinear velocity; VAP: average path velocity; VSL: straight linear velocity; STR: straightness; LIN: linearity (not significant). Control and 0.5 mg/mL, 1 mg/mL, 1.5 mg/mL, and 2 mg/mL myo-inositol-treated groups. Letters a, b, and c represent significant differences between groups (*p* < 0.05).

## Data Availability

The data presented in this study are available on request from the corresponding author.
